# Phase I/II clinical trial of dendritic-cell based immunotherapy (DCVAC/PCa) combined with chemotherapy in patients with metastatic, castration-resistant prostate cancer

**DOI:** 10.18632/oncotarget.4145

**Published:** 2015-05-29

**Authors:** Michal Podrazil, Rudolf Horvath, Etienne Becht, Daniela Rozkova, Pavla Bilkova, Klara Sochorova, Hana Hromadkova, Jana Kayserova, Katerina Vavrova, Jan Lastovicka, Petra Vrabcova, Katerina Kubackova, Zdenka Gasova, Ladislav Jarolim, Marek Babjuk, Radek Spisek, Jirina Bartunkova, Jitka Fucikova

**Affiliations:** ^1^ Department of Immunology, Charles University, 2nd Faculty of Medicine and University Hospital Motol, Prague, Czech Republic; ^2^ Sotio, Prague, Czech Republic; ^3^ Department of Oncology, Charles University, 2nd Faculty of Medicine and University Hospital Motol, Prague, Czech Republic; ^4^ Institut National de la Santé et de la Recherche Médicale (INSERM), Centre de Recherche des Cordeliers, Paris, France; ^5^ Université Pierre et Marie Curie-Paris, Paris, France; ^6^ Université Paris Descartes, Paris, France; ^7^ Department of Pediatric and Adult Rheumatology, University Hospital Motol, Prague, Czech Republic; ^8^ Institute of Hematology and Blood Transfusion, Prague, Czech Republic; ^9^ Department of Urology, Charles University, 2nd Faculty of Medicine and University Hospital Motol, Prague, Czech Republic

**Keywords:** immunotherapy, dendritic cell, prostate cancer, overall survival, castration-resistant prostate cancer

## Abstract

**Purpose:**

We conducted an open-label, single-arm Phase I/II clinical trial in metastatic CRPC (mCRPC) patients eligible for docetaxel combined with treatment with autologous mature dendritic cells (DCs) pulsed with killed LNCaP prostate cancer cells (DCVAC/PCa). The primary and secondary endpoints were safety and immune responses, respectively. Overall survival (OS), followed as a part of the safety evaluation, was compared to the predicted OS according to the Halabi and MSKCC nomograms.

**Experimental design:**

Twenty-five patients with progressive mCRPC were enrolled. Treatment comprised of initial 7 days administration of metronomic cyclophosphamide 50 mg p.o. DCVAC/PCa treatment consisted of a median twelve doses of 1 × 10^7^ dendritic cells per dose injected s.c. (Aldara creme was applied at the site of injection) during a one-year period. The initial 2 doses of DCVAC/PCa were administered at a 2-week interval, followed by the administration of docetaxel (75 mg/m2) and prednisone (5 mg twice daily) given every 3 weeks until toxicity or intolerance was observed. The DCVAC/PCa was then injected every 6 weeks up to the maximum number of doses manufactured from one leukapheresis.

**Results:**

No serious DCVAC/PCa-related adverse events have been reported. The median OS was 19 months, whereas the predicted median OS was 11.8 months with the Halabi nomogram and 13 months with the MSKCC nomogram. Kaplan-Meier analyses showed that patients had a lower risk of death compared with both MSKCC (Hazard Ratio 0.26, 95% CI: 0.13–0.51) and Halabi (Hazard Ratio 0.33, 95% CI: 0.17–0.63) predictions. We observed a significant decrease in Tregs in the peripheral blood. The long-term administration of DCVAC/PCa led to the induction and maintenance of PSA specific T cells. We did not identify any immunological parameter that significantly correlated with better OS.

**Conclusions:**

In patients with mCRPC, the combined chemoimmunotherapy with DCVAC/PCa and docetaxel was safe and resulted in longer than expected survival. Concomitant chemotherapy did not preclude the induction of specific anti-tumor cytotoxic T cells.

## INTRODUCTION

Prostate cancer (PCa) is the most frequently diagnosed noncutaneous malignancy in elderly men and is the second leading cause of cancer-related death in Western countries [1]. Localized, early-stage disease is, in general, successfully treated with surgery or radiation therapy; however, approximately 30% of patients have recurrence and require further management. Androgen deprivation is the standard of care in such situations, achieving temporary tumor control or regression in up to 85% of cases [2]. Although castration is quite effective, most patients ultimately develop progressive disease, which is poorly responsive to traditional therapies and remains a significant clinical challenge [3]. Since 2004, docetaxel-based regimens have become the first-line chemotherapy in metastatic, castration-resistant prostate cancer (mCRPC) patients [4–6]. Apart from taxanes, three additional agents that directly target tumor cells have recently been reported to increase the median OS in mCRPC patients as well: enzalutamide, an anti-androgen therapy [7]; abiraterone - an inhibitor of testosterone synthesis [8]; and Alpharadin, an alpha-emitter that targets bone metastasis [9]. However, these strategies only modestly prolong patient survival and are linked to a wide range of undesirable side effects [10].

Cancer immunotherapy is being tested as an additional treatment modality in oncology [11]. Sipuleucel-T (Provenge; Dendreon Corporation), a prostatic acid phosphatase-granulocyte/macrophage-colony-stimulating factor (PAP-GM-CSF) fusion protein-loaded autologous blood cell vaccine, was approved by the US Food and Drug Administration (FDA) for the treatment of asymptomatic or minimally symptomatic mCRPC [12].

Active cellular immunotherapy (ACI) using antigen-loaded dendritic cells (DCs) is another immunotherapeutic approach in the clinical development [13, 14]. Although many trials reported the induction of antitumor immune responses after administration of cancer immunotherapy, the efficacy has been disappointing. The limited success of ACI in advanced cancer patients might be due to the establishment of tumor-induced immunosuppression [15]. In such situation, immunotherapy alone cannot be expected to radically reverse the progressive course of the disease. Experimental evidence supports the fact that the goal of the immunotherapy in the late stages is not necessarily complete eradication tumor cells but rather the establishment of an equilibrium between the host immune system and the proliferating tumor cells [16]. Therefore, new directions have focused on combination strategies that could improve the vaccine efficacy without adding significant toxicity.

The concept of combined chemoimmunotherapy explores the fact that the treatment with chemotherapy might not only decrease the tumor cell load but also neutralize the tumor induced immunosuppression thus facilitating the effect of concurrent immunotherapy. In support of testing docetaxel in combination with a vaccine, experimental data obtained in mice and humans have contradicted the traditional thinking that taxanes suppress immune-cell functions [17, 18]. *In vitro* assays have revealed that a cohort of patients with stage II/III breast cancer had enhanced T cell and NK cell functions when treated with taxanes [19]. Docetaxel has also been shown to reverse myeloid derived suppressor cell-mediated immune suppression and to modulate the tumor microenvironment in a manner that improves the efficacy of immune-based therapies [20]. Moreover, patients previously vaccinated with an anti-cancer vaccine may respond longer to docetaxel compared with historical controls receiving docetaxel without prior immunotherapy [21].

In this Phase I/II trial, we tested the combined chemoimmunotherapy in patients with metastatic castration resistant prostate cancer. In addition to the standard chemotherapy, patients eligible for docetaxel were treated with autologous dendritic cell based vaccine, DCVAC/PCa. DCVAC/PCa is composed of autologous Poly I:C activated dendritic cells pulsed with killed LNCaP prostate cancer cell line.

## MATERIALS AND METHODS

### Patient eligibility

Eligible patients had prostatic adenocarcinoma and progression of PSA serum levels and/or radiographic progression after the failure of second-line hormonal manipulation in generalized, metastatic disease. Previous chemotherapy was allowed if the last dose was at least 3 months before the study entry. Other eligibility requirements were an Eastern Cooperative Oncology Group performance status (ECOG) of 0–2, adequate hematologic, hepatic, and renal function, and negative status for hepatitis B and C viruses and HIV. Exclusion criteria included a history of primary immunodeficiency, a severe allergic or anaphylactic reaction following vaccination, the presence of pulmonary, cardiac, or other systemic diseases limiting patient survival.

### Study design and treatment

**Figure 1:**
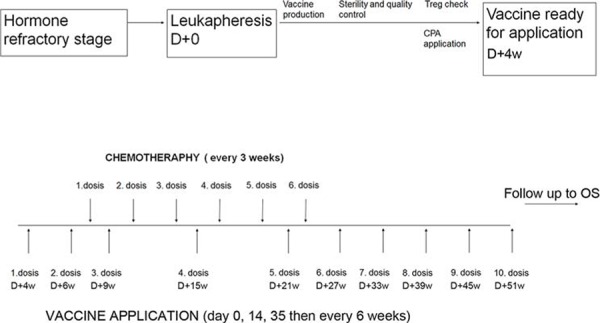
Study design The DCVAC/PCa treatment consisted of a median twelve doses of 1 × 10^7^ dendritic cells injected s.c. The treatment comprised an initial 7 days of metronomic cyclophosphamide administration 50 mg p.o. and 2 subsequent doses of DCVAC/PCa. Patients then started docetaxel (75 mg/m^2^) and prednisone (5 mg twice daily) treatment, which was administered every 3 weeks; DCVAC/PCa was then given every 6 weeks up to the maximum number of doses manufactured from one leukapheresis. Imiquimod 5% (Aldara® 5% drm cream) was applied locally 24 hours before and after each DCVAC/PCa administration. Immunomonitoring (IM) was evaluated after the first and twelfth doses of DCVAC/PCa. Clinical evaluation (CE) was performed after every single DCVAC/PCa dose.

This report includes summary data for 25 patients, including 15 patients from a single-institution, single-arm, open-label phase I/II clinical trial (EudraCT 2009-017295-24) and 10 patients from previous patient's named program (approved by University Hospital Motol IRB). These patients fulfilled the identical inclusion criteria and were treated by the analogous schedule which was later applied into the protocol of the clinical trial. The treatment schedule is summarized in Fig. 1. Briefly, DCVAC/PCa treatment consisted of a median of twelve doses of 1 × 10^7^ dendritic cells injected s.c. at axillary and inguinal area (2.5 ml at each site). The initial two doses of DCVAC/PCa were administered at a 2-week interval, followed by the administration of docetaxel (75 mg/m2) and prednisone (5 mg twice daily) given every 3 weeks until toxicity or intolerance was observed. The vaccine was then injected every 6 weeks up to the maximum number of doses manufactured from one leukapheresis. Minimal interval between chemotherapy administration and immunotherapy was 7 days. Immune monitoring was performed before the 1^st^ dose of DCVAC/PCa and after the 12^th^ dose or after last dose if less than 12 doses were manufactured from the leukapheresis. Before the 1^st^ DCVAC/PCa dose was administered, patients received metronomic cyclophosphamide (Cyclophosphamide Orion® 50 mg daily for 1 week) [22–24]. To increase the motility of the injected cells and to support the accumulation of local dendritic cells *in vivo*, imiquimod (Aldara® cream 12.5 mg) was applied locally 24 hours before and after each injection. The primary endpoints were the safety and feasibility of DCVAC/PCa active cellular immunotherapy in mCRPC patients; the secondary endpoint was the immune response. The study protocol was approved by the Institutional Review Board (IRB) and the State Institute for Drug Control (SUKL). Written informed consent was obtained from all patients before any study procedures were conducted.

### Assessment of clinical activity and toxicity

The patients were monitored at each visit by conducting a patient history and physical examination. The Medical Dictionary for Regulatory Activities (MedDRA, Version 15.1) was used for the coding of adverse events (AEs). All patients underwent relevant radiologic and laboratory tests, the Halabi and MSKCC predictions of survival, laboratory and clinical data related to the time of the 1^st^ DCVAC/PCa administration were used. The real OS was calculated from the 1^st^ DCVAC/PCa administration until death or until the data lock (information about all surviving patients were available at data lock). Even if not included in the study protocol as an endpoint, we evaluated PSA response as a part of laboratory monitoring. Serum PSA was measured every six weeks and a response (for patients with a baseline PSA level of at least 20 ng per milliliter) was defined as a reduction from baseline of at least 50 percent that was maintained for at least six weeks during the combined chemoimmunotherapy treatment.

### DCVAC/PCa production

#### Generation of DCs under GMP conditions

Leukapheresis was performed using a Cobe Spectra separator (Cobe BCT, Lakewood, CO, USA). All of the following operations were performed under Good Manufacturing Practice (GMP) conditions in the GMP facility of University Hospital Motol using the protocol for DC generation that was approved by the State Institute for Drug Control, as previously described [25, 26]. The leukapheretic product was diluted in PBS + 1 mM EDTA (Lonza, Verviers, Belgium), and mononuclear cells were separated by Ficoll-Paque Premium (GE Healthcare, Waukesha, WI, USA) gradient centrifugation. Collected mononuclear cells (PBMC) were washed in PBS + 1 mM EDTA (Lonza), resuspended in CellGro medium (CellGenix, Freiburg, Germany) and plated in triple flasks (Thermo Scientific, Waltham, MA, USA) at 1 × 10^6^ cells per cm^2^ of surface area. After 2 h, non-adherent cells were washed with PBS (Lonza). Adherent monocytes were cultured for 6 days in CellGro (CellGenix) medium with 20 ng/ml of IL-4 (Gentaur, Kampenhout, Belgium) and 500 U/ml of GM-CSF (Gentaur); fresh cytokines were added on day 3. Immature DCs were harvested on day 6, washed in PBS (Lonza) and resuspended in CellGro (CellGenix).

#### Loading of immature DCs with killed prostate cancer cells and maturation of DCs

The PSA-positive prostate cancer cell line LNCaP was obtained from the American Type Culture Collection and grown in UltraCULTURE (Lonza, Verviers, Belgium) supplemented with GlutaMax (Life Technologies, Carlsbad, CA) under GMP conditions. LNCaP cells were detached with 0.05% Trypsin-EDTA (Lonza), washed and killed by UV irradiation (312 nm for 10 min). Harvested immature DCs (day 6) were pulsed with tumor cells at a DC:tumor cell ratio of 5:1 for 4 h. Tumor cell-pulsed DCs were then matured with 25 μg/ml of Poly I:C (*Invivogen*) overnight. Mature DCs were harvested, resuspended in Cryostor CS10 (BioLife Solutions, Bothell, WA, USA) and stored in liquid nitrogen.

### Assessment of immunological parameters

#### Routine immunological testing

Serum levels of immunoglobulin G, A, and M and C-reactive protein (in g/L) were assessed by automated nephelometry using an Immage 800 Immunochemistry System (Beckman Coulter). Serum autoantibodies, ANCA, RF and anti-cardiolipin were detected using the ANA test (BioRad, Philadelphia, PA), ANCA test (Inova Diagnostics, San Diego, CA), and ACA test (Orgentec Diagnostic, Mainz, Germany), respectively. Lymphocyte subsets were enumerated by flow cytometry using FACS CANTO II (BD Bioscience, Franklin Lakes, NJ) and were subsequently analyzed using FlowJo software (Tree Star, Ashland, OR, USA). Monoclonal antibodies against CD3, CD4, CD8, CD16, CD19 and HLA-DR were purchased from BD Biosciences.

#### Detection of regulatory T cells

Regulatory T cells (Tregs) were identified by surface staining with anti-CD3 Alexa700 (Exbio, Vestec, Czech Republic), CD4 PC7 (eBioscience, San Diego, CA), CD8 PE-Dy590 (Exbio), CD25 PerCPCy5.5 and CD127 Alexa647 (BioLegend, San Diego, CA) antibodies, followed by fixation and permeabilization with a FoxP3 staining buffer set (eBioscience) and intracellular staining with anti-FoxP3 FITC (eBioscience), as previously described [27, 28]. All samples were processed and analyzed immediately after blood sampling on FACSAria™ (Becton Dickinson, Heidelberg, Germany) and analyzed using FlowJo software (Tree Star, Ashland, OR).

#### Detection of antigen-specific T cells against PSA, MAGE-A1 and MAGE-A3

For each patient, the frequency of antigen-specific T cells against tumor antigens (PSA, MAGE-A1, MAGE-A3) was measured by flow cytometry. Antigens were included in the testing based on the previous analysis of the expression of tumor associated antigens in LNCaP cell line using real-time quantitative PCR. Peripheral blood mononuclear cells (PBMCs) were incubated for 10 days in RPMI 1640 medium (Life Technologies) supplemented with 10% heat-inactivated pooled human AB serum, 100 U/ml penicillin, 2 mmol/l L-glutamine, non-essential amino acid mix and sodium pyruvate (all from Life Technologies), as well as with mixtures of overlapping peptides (PepMix; JPT Peptide Technologies, Berlin, Germany) that spanned the whole sequence of prostate specific antigen (PSA), melanoma-associated antigen 1 (MAGE-A1) and melanoma associated antigen 3 (MAGE-A3), each at a concentration of 1 ug/ml. On days 4 and 7, IL-2 was added (20 UI/ml; Gentaur, Kampenhout, Belgium). On day 9, PBMCs were restimulated for 12 hours with each peptide mixture mentioned above, and brefeldin (BioLegend, San Diego, CA, USA) was added after 4 hours of incubation. The cells were first stained with antibodies against CD3-PC5, CD4-PC7 (eBioscience), and CD8-PE-Dy590 (Exbio). Then, the Aqua Blue Live/Dead cell viability assay (Life Technologies) was used to measure the population of dead cells. Thereafter, the cells were fixed with Fixation/Permeabilization buffer (BD Bioscience) and permeabilized with Permeabilization buffer (BD Bioscience). Intracellular IFN-γ staining was performed with a FITC-conjugated antibody (BD Bioscience), and IL-2 staining was performed with an APC-conjugated antibody (BD Bioscience), according to the manufacturer's instructions. Stained cells were immediately measured using a BD LSR II flow cytometer (BD Biosciences) and data analysis was performed using FlowJo software (Tree Star) after the exclusion of Live/Dead-positive cells. IFN-γ secretion was only considered to be antigen specific if the frequency of IFN-γ-secreting T cells that responded to peptide-pulsed PBMCs was at least 2 times greater than the frequency of IFN-γ secretion in response to the negative control (unpulsed PBMCs).

#### Detection of tumor antigen-specific antibodies against PSA and MAGE-A3

The recombinant proteins PSA and MAGE-3 (Abnova, Taipei, Taiwan) were diluted in Carbonate Coating Buffer (Life Technologies) to a final concentration of 1 μg/ml and were adhered to 96-well plates overnight at 4°C. The plates were blocked for 1 hour with Assay Buffer (Life Technologies), and then human sera diluted to 1:50, 1:100 and 1:200 were incubated in the antigen-coated wells for 2 h. The plates were then incubated with secondary antibody (goat polyclonal antibody to human IgG; Abcam, Cambridge, UK) for 1 hour. TMB substrate (Life Technologies) was then added and incubated for 20 minutes. The reaction was stopped by adding Stop Solution (Invitrogen, Prague, Czech Republic), and the plates were immediately read at an absorbance of 450 nm. As a positive control, cytomegalovirus glycoprotein B was used. The cutoff value designating a positive reaction was assessed as the mean OD of 15 healthy control human sera (NHS) + 3SD.

### Statistical analysis

Group comparisons were performed using the GraphPad Prism software (GraphPad software, La Jolla, CA). The effect of the treatment on immune parameters was assessed using the Wilcoxon signed-rank test. Scatterplots feature least square linear regressions lines. Survival analysis was performed using the R-package ‘survival.’ The log-rank test was used to compare the survival of treated patients to their expected survival using the Halabi [29] or Memorial Sloan Kettering Cancer Center (MSKCC) nomograms [30]. Cox proportional hazards models were used to assess univariate and multivariate associations between clinical variables and prognosis. Variables that were significantly associated with prognosis in the univariate analysis were further included in the multivariate analysis.

## RESULTS

### Characteristics of the patients

Between August 2008 and March 2014, twenty-five patients were treated with DCVAC/PCa. The median age at the start of immunotherapy was 73 years (age range 48–82 years), 88% of the patients had tumors with Gleason score ≥ 7 and none of them had signs of visceral disease. The median entry levels of prostate specific antigen (PSA) were 186 ng/mL (range 1–749 ng/mL), of lactate dehydrogenase (LDH) were 234 IU/L (range 129–399 IU/L), of alkaline phosphatase (ALP) were 192 IU/L (range 37–1843 IU/L), of hemoglobin (Hgb) were 11.9 g/dL (range 9–14.8 g/dL) and of C-reactive protein (CRP) were 5.6 mg/L. All patients had experienced progression on androgen deprivation therapy as an initial or secondary treatment. Testosterone levels were maintained at castrate levels during the study. Eight patients had received docetaxel-based chemotherapy prior to the enrollment (> 3 months before entering the study), with a median of 8 months long chemotherapy free period. During the study period, approximately 350 doses of ACI were administered, with a median of 12 doses per patient. After chemotherapy failure, the patients were treated with supportive care. None received abiraterone, enzalutamide or alpharadin. The patients' baseline characteristics are shown in Table 1.

**Table 1:** Patients' baseline characteristics

Patient characteristics	
Total number of patients	25
**Race**	
Caucasian	25
**Age (years)**	
Median	73
Mean	67
Range	48–82
**ECOG performance status**	
0	8
1	15
2	2
**Disease location**	
Bone only	13
Nodal only	4
Bone and nodal	8
**Gleason score**	
5	1
6	2
7	10
8	3
9	9
Median	7
**PSA, ng/mL**	
Median	186
Mean	245
Range	1–749
**Lactate dehydrogenase, IU/L**	
Median	234
Mean	248
Range	129–399
**Alkaline phosphatase, IU/L**	
Median	192
Mean	327
Range	37–1843
**Hemoglobin, g/dL**	
Median	11, 9
Mean	11, 9
Range	9–14, 8

Abbreviations: ECOG, Eastern Cooperative Oncology Group; PSA, prostate specific antigen.

### Adverse events

The overall toxicities are summarized in Table 2. During the administration of cumulative 350 doses, we recorded following adverse events (AEs): 17x fatigue, 13x back pain, 5x diarrhea, 3x constipation and 13x other gastrointestinal discomfort, 12x paresthesias, 8x mild infections, 3x loss of appetite, 1x hypersensitivity-like reaction and 1x myalgia (number of events). All AEs were grade 1 or 2, and there were no grade 4 toxicities or treatment-related deaths. None of the 44 reported serious adverse events (SAEs) were related to the immunotherapy but rather were related to the progression of the underlying disease or the concomitant chemotherapy. In addition, no suspected unexpected serious adverse reactions (SUSARs) were reported. In summary, DCVAC/PCa therapy was well tolerated with the favorable safety profile.

**Table 2:** Cumulative summary tabulation of serious adverse events (SAEs) The Medical Dictionary for Regulatory Activities (MedDRA) version 15.1 was used for the coding of adverse events (AEs). The summary tabulations of SAEs are arranged by the primary System Organ Class (SOC) and Preferred Term (PT) level.

System organ class	Active study drug (DCVAC/PCa)
Preferred term	
**Blood and lymphatic system disorders**	
Anaemia	4
Bone marrow failure	1
Febrile neutropenia	2
Leukopenia	1
Pancytopenia	1
Thrombocytopenia	1
**Cardiac disorders**	
Myocardial infarction	1
Pulmonary oedema	1
**General disorders and administration site conditions**	
Death	2
**Immune system disorders**	
Hypogammaglobulinaemia	1
**Injury, poisoning and procedural complications**	
Myopathy toxic	1
Spinal compression fracture	1
**Metabolism and nutrition disorders**	
Diabetes mellitus	1
Hypokalaemia	1
**Musculoskeletal and connective tissue disorders**	
Pain in extremity	1
Osteonecrosis of jaw	1
Pathological fracture	1
**Neoplasms benign, malignant and unspecified (incl cysts and polyps)**	
Choroid melanoma	1
Meningioma	1
Neuroendocrine carcinoma	1
**Nervous system disorders**	
Cognitive disorder	1
Epilepsy	1
Hemiparesis	1
Paraparesis	1
Paraplegia	1
**Renal and urinary disorders**	
Hydronephrosis	1
Incontinence	1
Renal failure	1
Urinary retention	2
Urinary tract inflammation	1
Urinary tract obstruction	2
**Respiratory, thoracic and mediastinal disorders**	
Pulmonary embolism	1
**Vascular disorders**	
Circulatory collapse	1
Hypotension	1
Thrombosis	3
**TOTAL**	**44**

### Clinical efficacy

#### PSA response

In patients with PSA response assessed, reduction by at least 50% on two visits at least 6 weeks apart was observed in 9 of 23 patients (39, 1%) (8/9 were chemo-naive). At 6 months (6^th^ dose of DCVAC/PCa) after the initiation of chemo/immunotherapy, a ≥ 50% decrease in PSA was observed in 8 of 23 patients (34, 8%) and 25–50% decrease of PSA in additional 5 patients (21, 7%).

#### Overall survival

**Figure 2:**
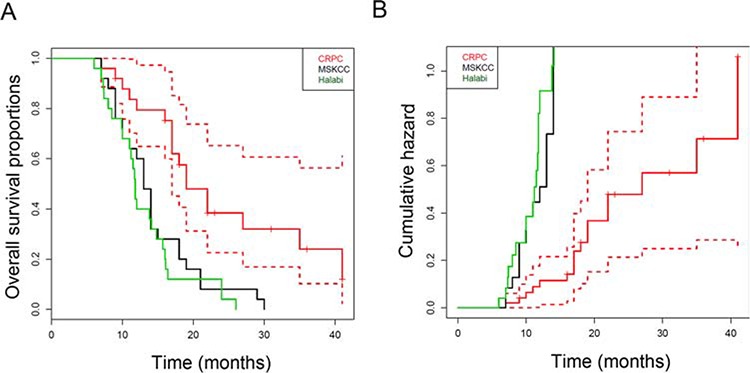
Overall survival of docetaxel and DCVAC/PCa treated patients (*n* = 25) **A.** Kaplan-Meier curves for overall survival and **B.** the cumulative hazard values of DCVAC/PCa-treated patients and the corresponding expected survival and hazard values predicted using the Halabi and MSKCC nomograms. The median overall survival was 19 months with DCVAC/PCa vs 11.8 months (Halabi) or 13 months (MSKCC) predicted by the nomograms, *p = 0.00005.

Overall survival, followed as a part of the safety evaluation, was compared to the predicted values calculated by the Halabi and MSKCC nomograms. Fig. 2A shows Kaplan-Meier estimation of the survival distributions. The estimated median survival for the DCVAC/PCa-treated group was 19 months compared to 11.8 months in the Halabi and 13 months in the MSKCC control predictions. Log-rank tests showed that patients had a significantly better observed survival than that of the MSKCC (*p = 0.0008) and Halabi (*p = 0.0001) predictions. Univariate Cox regressions showed that patients had a lower risk of death compared with both MSKCC (Hazard Ratio 0.26, 95% CI: 0.13–0.51) and Halabi (Hazard Ratio 0.33, 95% CI: 0.17–0.63) predictions (Fig. 2B).

### Immunological response

**Figure 3:**
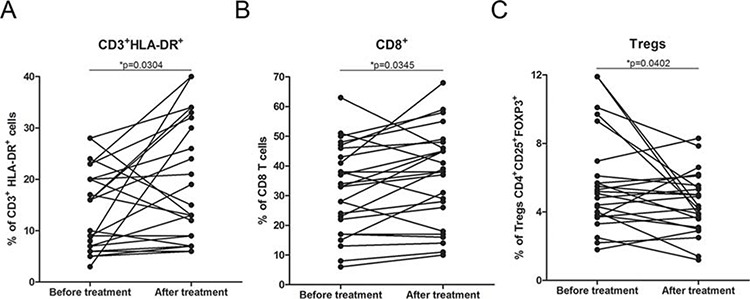
Immune parameters in the peripheral blood during DCVAC/PCa/docetaxel treatment **A.** The proportions of CD3^+^/HLA-DR^+^ and **B.** CD8^+^ cells were significantly increased after the treatment in the 25 evaluated patients, *p < 0.05. Data are expressed as the proportion of CD3^+^/HLA-DR^+^ and CD8^+^ cells among CD45^+^ cells. **C.** The percentage of regulatory T cells (CD4^+^ CD25^+^ FoxP3^+^) was significantly decreased after the treatment, *p < 0.05. Data are expressed as the proportion of CD4^+^ CD25^+^ FoxP3^+^ Tregs among CD4^+^ T cells.

To evaluate the effect of DCVAC/PCa on the immune system, PBMCs were isolated pre and post vaccination and subsequently analyzed for T cell subsets. We observed no significant changes in the frequency or absolute numbers of peripheral blood CD3^+^, CD4^+^ and NK cells during the course of the trial (Fig. S1). Conversely, the frequency of activated CD3^+^/HLA-DR^+^ cells and CD8^+^ T cells significantly increased (*p > 0.05) (Fig. 3A, 3B). Additionally, a significant decrease in the frequency of regulatory T cells was observed (*p = 0.0402) (Fig. 3C). Furthermore, after the course of treatment, the levels of IgG and IgM were significantly decreased (Supplemental Fig. 2A, 2B). There was no significant trend in the occurrence of autoantibodies (data not shown).

**Figure 4:**
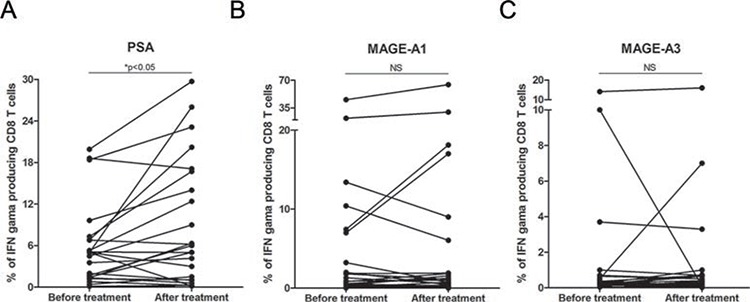
Tumor antigen-specific T cell response during DCVAC/PCa/docetaxel treatment in the peripheral blood **A.** The increase in the frequency of PSA-specific T cells, *p < 0.05, as well as the maintenance of stable levels of T cells specific against MAGE-A1 **B.** and MAGE-A3 **C.** was detected.

We also assessed the presence of antigen-specific T cells and antibody response against prostate-specific tumor antigens. The peripheral blood of the patients was stimulated by peptide mixes (PSA, MAGE-A1 and MAGE-A3), and the frequency of IFN-γ-secreting T cells was analyzed by flow cytometry. Eleven out of 23 patients had significantly higher numbers of antigen-specific T cells against PSA before treatment compared with healthy controls (data not shown). Similar results were obtained for MAGE-A1 and MAGE-A3 antigen-specific T cells, for which 6 out of 23 and 3 out of 23 patients had significantly increased numbers of antigen-specific T cells compared with the healthy controls (data not shown). Long-term administration of DCVAC/PCa induced a statistically significant increase in the PSA-specific T cells PSA (*p < 0.05) (Fig. 4A). However, we did not observe significant changes in the frequency of antigen-specific T cells against MAGE-A1 and MAGE-A3 antigens during the course of the trial (Fig. 4B, 4C).

Furthermore, we evaluated the induction of tumor antigen-reactive IgG antibodies by DCVAC/PCa vaccination. The presence of IgG antibodies against PSA and MAGE-3 was analyzed in patient sera. We detected IgG-positive antibodies against PSA in 6 out of 23 (26%) patients (Supplemental Fig. 2C) and against MAGE-A3 in 8 out of 23 (34%) patients (Supplemental Fig. 2D). There was no obvious correlation between the presence of PSA or MAGE-A3 specific antibodies and frequency of tumor antigen specific T cells (Supplemental Fig. 3A, 3B). We did not observe any significant correlation between the OS and the presence of antibody or cellular immunity against tumor antigens (Supplemental Fig. 3C, 3D, 3E, 3F, 3G).

### Cox proportional hazards regression

Cox proportional hazards regression analysis was performed to determine factors that could predict disease progression or death. First, univariate analysis was performed to evaluate the impact of 24 biological parameters measured at the beginning of the treatment (Table 3). Among those, C-reactive protein (CRP) was associated with a poor outcome after treatment (Hazard Ratio: 1.01), whereas ECOG and hemoglobin (Hgb) were associated with a favorable outcome after treatment (Hazard Ratio: 0.89 and 0.64, respectively). No other parameters reached significance. Multivariate analysis indicated that Hgb (Hazard ratio 0.68, 95% CI: 0.48–0.95) was the only independent prognostic factor associated with a positive outcome of treated patients, although the performance status of ECOG was close to being significantly associated with a good prognosis (Hazard ratio 0.91, 95% CI 0.99–7.1, *p*-value 0.052). None of the routine immunological parameters evaluated before treatment had any impact on the overall survival of vaccinated subjects.

**Table 3:** Cox proportional hazards regression analysis of the association between potential factors and death after DCVAC/PCa in mCRPC patients

	Univariate				Multivariate			
Factor	95% Confidence Interval				95% Confidence Interval			
	Hazard Ratio	*p*-value	Lower bound	Upper bound	Hazard Ratio	*p*-value	Lower bound	Upper bound
ECOG	0.89	0.0059	0.81	0.96	0.91	0.05	0.99	7.1
Hemoglobin	0.65	0.0046	0.47	0.87	0.68	0.02	0.48	0.95
CRP	1.01	0.04	1.00	1.03	1.00	0.76	0.99	1.02
Pts. age	0.99	0.95	0.92	1.08	-	-	-	-
iPSA	0.99	0.48	0.99	1.00	-	-	-	-
PSA	1.00	0.19	0.99	1.00	-	-	-	-
Gleason score	0.87	0.55	0.54	1.39	-	-	-	-
IgG	1.13	0.21	0.93	1.37	-	-	-	-
IgA	1.54	0.18	0.81	2.92	-	-	-	-
IgM	0.73	0.59	0.23	2.33	-	-	-	-
LE	0.95	0.55	0.79	1.13	-	-	-	-
LY	0.86	0.95	0.004	180.2	-	-	-	-
T lymphocytes (CD3)	1.00	0.73	0.97	1.03	-	-	-	-
CD3^+^ HLADR^+^	0.98	0.55	0.92	1.04	-	-	-	-
CD3^+^ CD16^+^ cells	0.99	0.86	0.96	1.03	-	-	-	-
CD4^+^ T cells	1.01	0.6	0.96	1.06	-	-	-	-
CD8^+^ T cells	1.00	0.9	0.97	1.03	-	-	-	-
B Lymphocytes	0.98	0.64	0, 9	1.06	-	-	-	-
Treg	0.84	0.1	0.67	1.03	-	-	-	-
Alkaline phosphatase	1.17	0.07	0.91	1.39	-	-	-	-
LDH	1.77	0.14	0.82	3.77	-	-	-	-
PSA specific T cells	1.06	0.27	0.95	1.17	-	-	-	-
MAGE-A1 specific T cells	1.01	0.48	0.97	1.05	-	-	-	-
MAGE-A3 specific T cells	1.00	0.97	0.87	1.15	-	-	-	-

Abbreviations: ECOG, Eastern Cooperative Oncology Group; CRP, C-reactive protein; iPSA, initial PSA; LE, leukocytes; ly, Lymphocytes; Treg, regulatory T cells; LDH, lactate dehydrogenate

## DISCUSSION

Prostate cancer represents a relevant candidate disease for the development of cancer immunotherapy strategies. Prostate cancer cells express tissue-specific proteins that could act as therapeutic targets, among others PSA, PAP, PSMA and prostate cancer usually progresses at relatively slow pace, which might allow for the elicitation of an effective immune response [31]. DC-based vaccination strategies have shown promising results in the past; however, the limitations observed in late-stage cancer patients led to the idea that combination strategies might improve the efficacy and long-term effects of immunotherapy [32, 33]. Experimental evidence supports the fact that the goal of immunotherapy in advanced-stage cancer does not have to be the complete eradication of tumor cells but rather the reversal from the escape phase back to the equilibrium stage as predicted by the cancer immunoediting model [34]. A plausible strategy for testing cancer immunotherapy would be to design trials in early stages of the disease, with minimal burden of tumor cells [35, 36]. It is, however, very challenging to define studies in early stage patients with efficacy indicating endpoints that could be reached in a realistic timeframe. Current regulatory environment pushes for the improved overall survival as the most relevant indicator of a clinical benefit. Cancer immunotherapy approaches thus need to be tested in late stage patients, often pretreated or treated by chemotherapy with large tumor burden and metastatic disease. It's very challenging to induce anti-tumor responses in late stages in the settings of a profound tumor induced immunosuppression. Possible strategy how to circumvent these practical concerns might be the appropriate combination of tumor mass reduction by surgery or chemo/radiotherapy along with the neutralization of tumor-induced immunosuppression [17]. This might establish the proper conditions for the induction of an anti-tumor immune response by active immunotherapy.

Recent studies indicate that despite the common view of chemotherapy and immunotherapy as antagonistic, there are synergies between the two approaches. For example, certain chemotherapeutics were described to induce immunogenic cell death [37–40]. Chemotherapy can also reduce tumor induced immunosuppression by eliminating suppressive populations of immune cells, such as Tregs or myeloid derived suppressor cells [37]. Immunotherapy has been reported to sensitize tumor cells to subsequent chemotherapy in various models, including small cell lung cancer or glioblastoma [41] [42].

With respect to the concept of combined chemoimmunotherapy, we performed an open-label, single-arm clinical trial in patients with metastatic, castration-resistant prostate cancer (mCRPC) eligible for first- or second-line docetaxel treatment using DCVAC/PCa. We did not see any serious anaphylactic reactions or any evidence of autoimmunity in treated subjects. Our data are consistent with the published reports, showing a favourable safety profile of DC-based approaches [32]. Moreover, no additional toxicity of combining chemotherapy with vaccination has been observed. Importantly, patients receiving combined treatment with standard docetaxel chemotherapy and DCVAC/PCa survived significantly longer than predicted by standard Halabi and MSKCC nomograms. With a median follow-up of 19 months, combined docetaxel and DCVAC/PCa resulted in a 7.2- and 6-month improvement in the median overall survival compared with that of the Halabi or MSKCC nomogram, respectively. The effect of on survival was consistently observed across patient subgroups, including those with prognostic factors known to be adversely correlated with overall survival such as increased prostate specific antigen, alkaline phosphatase and lactate dehydrogenase levels, the Gleason score, the presence of pain, and an increased number of bone metastases. Our results showed that factors reflecting satisfactory clinical condition of the treated patients (good performance status, low CRP levels, higher Hgb levels) were associated with longer overall survival. None of the assessed routine immunological parameters had an important predictive value for the outcome of the therapy. This is in contrast with the data published by Sheikh N et al [12].

In a pivotal clinical trial with docetaxel (5), rates of PSA response were detected in 45% of docetaxel treated patients. In this study, we detected PSA response in 39, 1% patients. However, 9 patients in our study were advanced patients who previously failed on docetaxel before the study entry. When restricting the analysis of the PSA response to chemo-naive patients, the PSA response was detected in 60%. This suggests that combined chemo-immunotherapy might lead to the PSA response in higher proportion of patients than docetaxel alone. This, however, needs to be analyzed in larger randomized trials.

Immune responses were evaluated as a secondary endpoint in our study. We evaluated antigen-specific CD8^+^ T cell responses by intracellular staining for IFN-γ following stimulation with tumor antigens and antibody responses by measuring tumor antigen specific IgGs. We detected a significant increase in the frequency of PSA-specific CD8^+^ T cells, with no increase in the number of antigen-specific T cells against MAGE-A1 and MAGE-A3. The total number of activated CD3^+^HLADR^+^ T cells, as well as of cytotoxic CD8^+^ T cells, was significantly increased after the treatment cycle.

In accordance with previously published reports, we detected lower levels of total IgG and IgM in the sera after the treatment. The decline is most probably linked to the chemotherapy treatment [43]. In 6 and 8 patients' sera, we detected the presence of IgG antibodies against PSA and MAGE-A3, respectively. There was no direct correlation between patients with antigen-specific T cells and positive antibodies against the respective tumor antigen. We did not see any correlation between the presence of anti-tumor immune response and survival, although this analysis is preliminary given the small number of patients. It's also important to note that although easily accessible, peripheral blood might not represent the most relevant compartment for the analysis of anti-tumor immunity. Analysis of the tumor microenvironment might provide more pertinent information.

Prostate cancer patients were reported to have increased numbers of circulating and tumor-infiltrating Tregs, and there is evidence that Tregs promote tumor growth *in vivo* [44]. We detected significantly lower frequency of Tregs after chemo-immunotherapy.

Taken together, it's hard to dissect which changes in immune parameters are attributable to chemo-immunotherapy and which are caused by the disease progression. We conclude that chemotherapy does not preclude the induction and long term maintenance of PSA-specific T cells and that DCVAC/PCa does not induce detectable autoimmunity. The observed changes in immune parameters (reduction of Tregs, increase in CD8+ T cells, HLA DR^+^CD3^+^ cells and PSA-specific CD8^+^ cells) fit into the concept that successful cancer immunotherapy should not increase the percentage of regulatory T cells and should lead to the establishment of a long-term tumor cell-specific immunity [45].

Combined chemoimmunotherapy by docetaxel and DCVAC/PCa was well tolerated and led to an improved overall survival of mCRPC patients compared with the predicted survival using the nomograms [29, 30]. Better than predicted survival, favorable safety profile, augmentation of the antigen-specific immune responses and decrease in Treg numbers provide rationale for conducting larger randomized studies, including placebo-control group, to evaluate the clinical efficacy of this treatment strategy.

## SUPPLEMENTARY FIGURES


