# Supplementary material for: Phase I/II clinical trial of dendritic-cell based immunotherapy (DCVAC/PCa) combined with chemotherapy in patients with metastatic, castration-resistant prostate cancer
Source: Oncotarget. 2015 May 29;6(20):18192–205. doi: 10.18632/oncotarget.4145 (PMC4627245; doi:10.18632/oncotarget.4145)
Supplement: Supplementary file 1 [file oncotarget-06-18192-s001.pdf]

SUPPLEMENTARY FIGURES

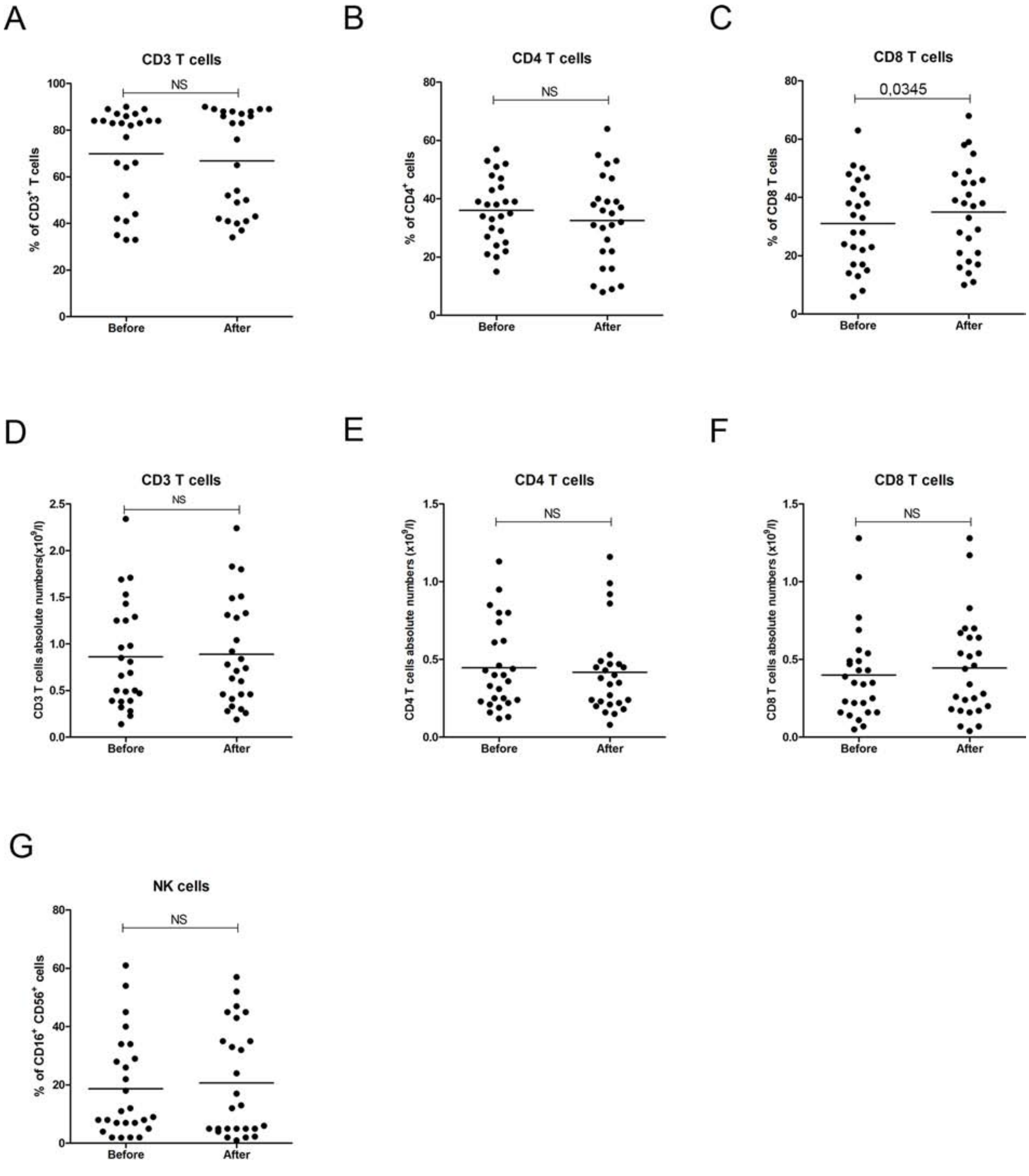

Supplementary Figure S1: Immune parameters in the peripheral blood during DCVAC/PCa/docetaxel treatment. Proportions of CD3 **A, D.** CD4 **B, E.** CD8 cells **C, F.** and NK cells **G.** remained unchanged after the treatment in 25 patients. Data are expressed as the proportions of CD3, CD4, CD8 and NK cells among $CD45^{+}$ cells A, B, C, G and as absolute numbers D, E, F.

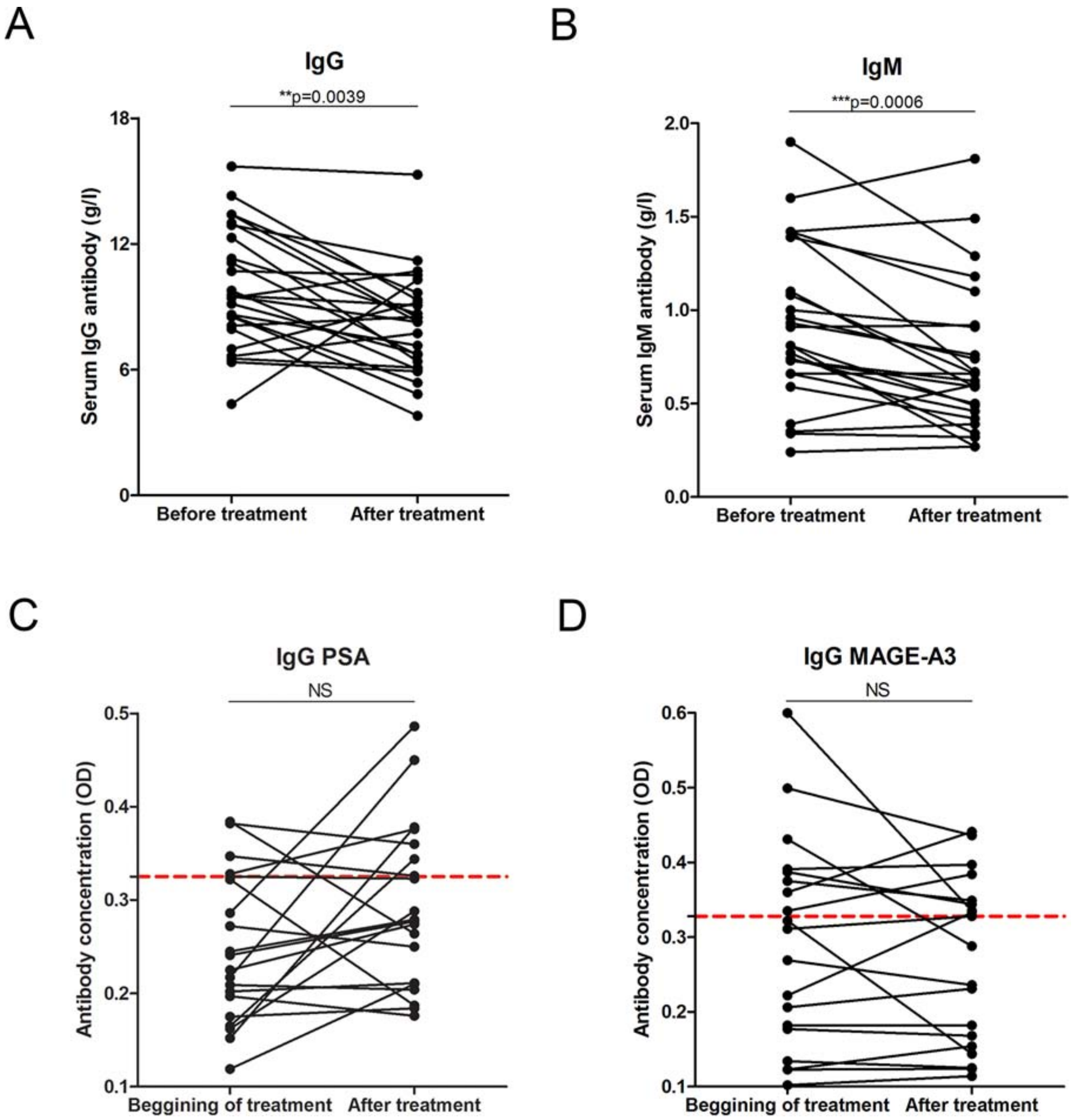

Supplementary Figure S2: Humoral immune response in patient's serum including the tumor antigen-specific response during DCVAC/PCa/docetaxel treatment. The serum concentrations of IgG **A.** and IgM **B.** were significantly lower after the treatment, $^{**}p < 0.001$ and $^{***}p < 0.0001$, respectively. Concentrations of IgG antibodies against PSA **C.** and MAGE-A3 **D.** were measured in the patients' sera. The cutoff value (red line) designating a positive reaction was calculated as the mean OD of the 15 healthy control human sera + 3SD.

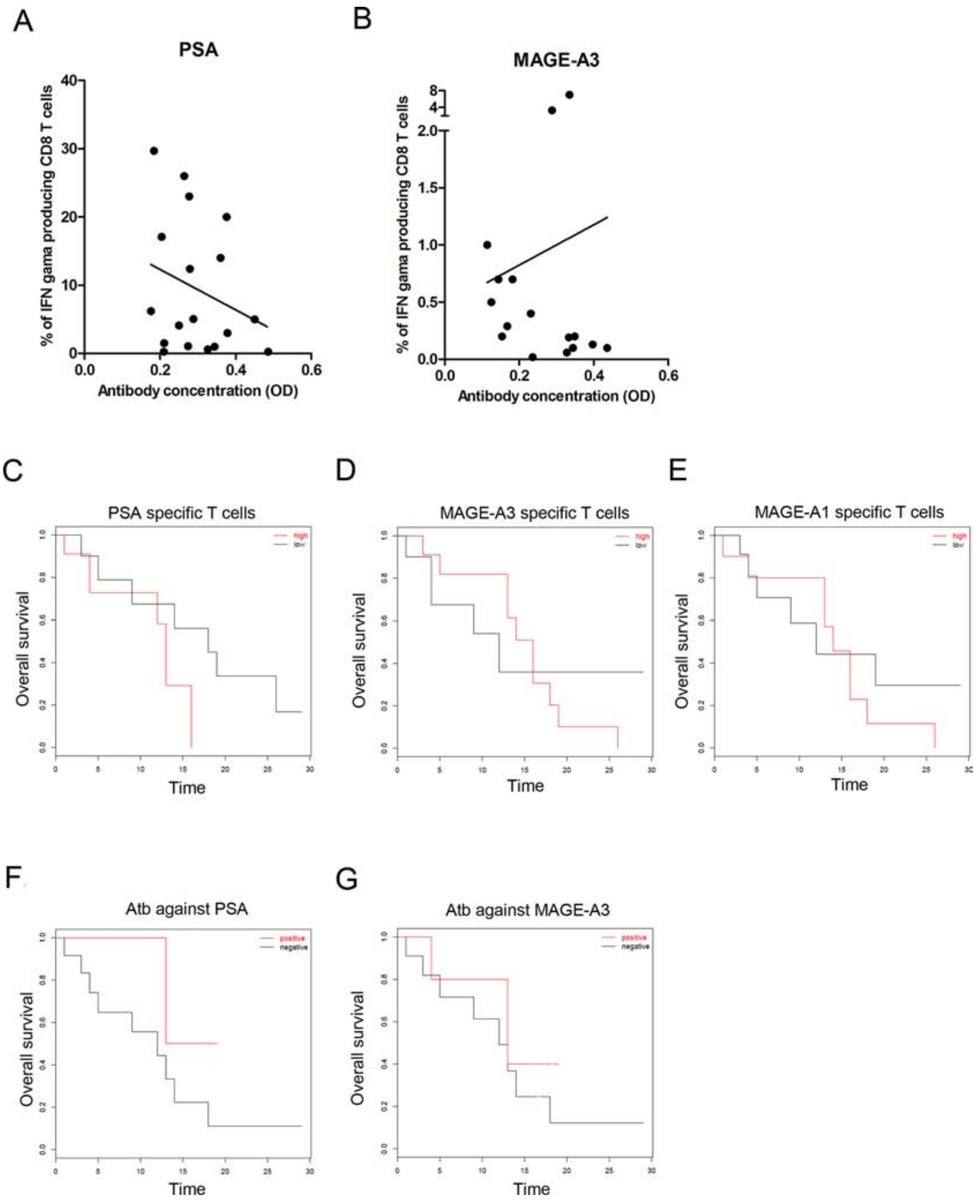

Supplementary Figure S3: Correlation between the frequency of antigen-specific T cells against PSA A. and MAGE-A3 **B.** and antibody titers (OD) detected in the peripheral blood of 25 patients. Kaplan-Meier curves representing the relationship between immune parameters and overall survival after DCVAC/PCa treatment. Patients were stratified according to the median value of **C.** PSA-specific T cells, **D.** MAGE-A3-specific T cells and **E.** MAGE-A1-specific T cells or serum immunoreactivity against **F.** PSA and **G.** MAGE-A3. All log-rank tests were non-significant.
